# An Epidemiological Survey Regarding Ticks and Tick-Borne Diseases among Livestock Owners in Punjab, Pakistan: A One Health Context

**DOI:** 10.3390/pathogens10030361

**Published:** 2021-03-18

**Authors:** Sabir Hussain, Abrar Hussain, Jeffery Ho, Jun Li, David George, Abdul Rehman, Jehan Zeb, Olivier Sparagano

**Affiliations:** 1 Department of Infectious Diseases and Public Health, Jockey Club College of Veterinary Medicine and Life Sciences, City University of Hong Kong, Kowloon, Hong Kong, China; HO.jeff@connect.PolyU.hk (J.H.); jun.li@cityu.edu.hk (J.L.); 2 Department of Epidemiology and Public Health, University of Veterinary and Animal Sciences, Lahore 54600, Pakistan; abrar.arid@gmail.com (A.H.); abdul.rehman@uvas.edu.pk (A.R.); 3 School of Data Science, City University of Hong Kong, Kowloon, Hong Kong, China; 4 School of Natural and Environmental Sciences, Agriculture Building, Newcastle University, Newcastle upon Tyne NE1 7RU, UK; david.george1@newcastle.ac.uk; 5 Department of Zoology, Abdul Wali Khan University Mardan, Mardan 23200, Pakistan; zebjehan2012@gmail.com

**Keywords:** livestock, tick-borne disease, zoonosis, environment, one health

## Abstract

Recent global changes have led to an increase in the spread of ticks and tick-borne diseases (TBDs) affecting domestic ruminants and humans, with an annual loss of US $13.9–$18.7 billion. The current study determined the perception and practices of livestock farmers regarding tick infestation. A total of 112 livestock farms were surveyed in Punjab, Pakistan, among which animals from 42 (37.5%) farms were infested with ticks. Only 28.6% (*n* = 32) of the dairy farmers were consulting veterinarians for ticks control, while 86.7% (*n* = 97) of the respondents did not consider biosecurity measures in the control of tick transmission. Most of the respondents, 71.4% (*n* = 80), did not consider manual tick removal from their animals (i.e., by hand, followed by physically crushing) as a risky practice for spreading zoonotic diseases. Improper disposal of bottles of acaricides in the farm drainage was also observed, putting the environment and aquatic life at risk. These wrong practices may contribute to high disease burdens and economic losses, increasing the possibility of transmission of zoonotic TBDs and pollution of the environment. Therefore, an integrated One Health approach is required for the control of TBDs through environmentally friendly approaches.

## 1. Introduction

Pakistan is an agricultural hub, and domestic animals are the major contributor of Pakistan’s economy. Ectoparasites are major factors for decrease in farm animal production [1]. Among these ectoparasites, ticks are a major concern in the livestock sector of Pakistan. Ticks are hematophagous arachnid ectoparasites that feed on the blood of many animals, including humans [2]. Three key economically important tick families have been classified, namely *Ixodidae, Argasidae*, and *Nuttalliellidae*. The *Ixodidae* is comprised of 949 known species, *Argasidae* has 200, and *Nuttalliellidae* has only 1 species. Ticks suck blood from their host, which they locate by responding to cues associated with host odors, breath, body heat, and the vibration of the victim. Ticks are usually found on the udder, ear, groin region, and tails of cattle [3], where they can affect livestock directly by causing irritation and allergic reaction [4,5]. Ticks are also one of the most important biological disease vectors in the environments where they are found, posing a threat to both animal and human health alike. 

Ticks cause negative impacts on human and animal health through infestation and transmission of a wide range of pathogens, including viral, bacterial, and protozoal diseases [5]. Ticks and the pathogens they transmit are a growing burden on human and animal health world-wide. To date, several studies from Pakistan reported that more than 80% of bovines were tick-infested with species of *Hyalomma* and *Rhipicephalus* [6,7,8,9], which transmit tick-borne pathogens (TBPs) causing babesiosis, theileriosis, and anaplasmosis in ruminants and Crimean Congo haemorrhagic fever (CCHF) in humans [10,11,12,13,14,15]. Humans are infected by tick-borne diseases (TBDs) in many ways, such as via biting of ticks, and/or contact with blood or tissue of the infected animal. As ticks externally attached to their hosts, people involved in livestock handling, including slaughterhouse workers, veterinarians, laborers , laboratory workers, and milkmen, are at high risk of being infected [16,17]. Tick-borne diseases such as Lyme disease, rickettsiosis, CCHF, and tick-borne encephalitis are present in humans in all over the world, including Pakistan [18,19,20,21,22,23,24,25,26,27,28]. Of the approximately 949 identified tick species, circa 10% are vectors of TBPs, including agents of various infectious diseases having profound public health importance [5,13]. These ticks’ saliva may also cause skin lesions and systemic reactions in humans [29,30,31,32]. Several biological and chemical methods have been used to control ticks, but these have proved ineffective and unsatisfactory, mainly due to the development of acaricide resistance in many species [33,34], undesirable non-target toxicity [35,36,37], and the prohibitive costs of chemical tick control treatments [38]. Most of the studies conducted in Pakistan referred to the identification of the adult stages of ticks by a morphological (phenetic) method. However, species identification by morphological means can be challenging, particularly in diagnostic laboratories with limited entomological expertise [39,40]

Climate and environmental changes in Pakistan are likely to increase the tick abundance which will, in turn, increase the risk of human exposure to these arthropods and the incidence of human infections with TBPs [41,42,43,44,45,46]. Tick control in Pakistan is also challenging particularly due to import of exotic breeds of cattle, which is more prone to tick infestation under Pakistan’s climatic conditions [47], and the ability of ticks to quickly spread over large areas through feeding on migrating hosts, coupled with their ability to readily adapt to new habitat conditions [48,49].

Despite the above, few studies have been conducted and published on people’s knowledge, attitude, and practices regarding tick and TBDs, with even fewer from Pakistan, where TBDs are causing devastating economic losses [50,51,52,53,54,55,56]. With this in mind, the current study was designed to evaluate livestock owner’s knowledge, attitude, and practices towards ticks in Punjab, Pakistan, to assess the awareness about ticks and TBDs, zoonotic concerns, and need for a One Health approach towards tick control in this region.

## 2. Results

### 2.1. Prevalence of Ticks

**Figure 1:**
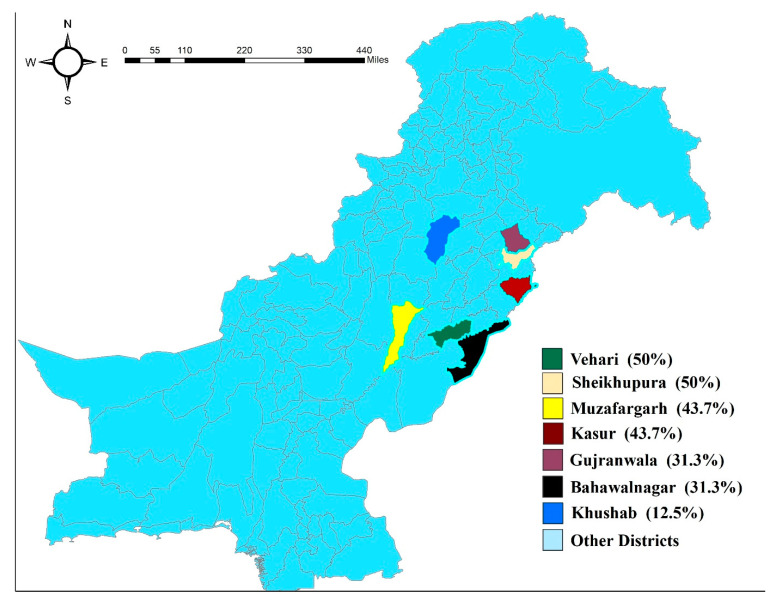
Map to show the location of study regions in Punjab, Pakistan. District-wise herd-based prevalence of tick infestation is also shown.

We calculated herd prevalence of tick infestation with the criteria of minimum five tick infested animals on the farm. Based on that criteria, 42 (37.5%) farms out of 112 overall were found to be positive for tick infestation. The highest tick prevalence observed was in the Sheikhpura and Vehari districts (50%), followed by Kasur and Muzaffargarh (43.7%). In comparison, the lowest prevalence was seen in Khushab (12.5%), followed by the Gujranwala and Bahawalnagar districts (31.3%) (Figure 1).

### 2.2. Production Characteristics and Perceptions about Tick Infestation

Fifty-three (47.3%) farmers kept their animals on the farm, while 59 (52.7%) kept them in their homes. Of the 112 farms surveyed, 29 (25.9%) were located near marshy areas. Twenty-eight (25%) farmers used stall feeding method, while 24 (21.4%) were practicing grazing, however 60 (53.6%) farmers adopted both the feeding strategies. Regarding ticks and TBDs, 64 (57.1%) farmers were aware of the risk factors associated with tick infestation in animals, while 53 livestock owners (47.3%) had knowledge of TBDs and recognized sandy floors as a risk factor for tick presence. A large percentage of the respondents (40.2%; i.e., 45 farmers) considered summer as the most prevalent season for tick infestation, but other seasons were also noted. Regarding tick control measures, 39 farms (34.8%) used herbal-based or traditional treatments, 24 (21.4%) ignored the problem, and only 32 (28.6%) consulted a veterinarian. Seventeen respondents (15.2%) reported that they sold infested animals as a means of controlling tick numbers on their own farms. The average cost of the acaricide per animal per year was reported among 104 respondents as PKR (Pakistani rupees) 4535 (28.87 USD, on 13 March 2021) with the range of 1000–9500 PKR, while 8 respondents never used acaracides. While average frequency of using acaricides on farm per year among 104 respondents was four with the range of 1–11 (Table 1).

### 2.3. Practices of Acaricidal Use

In our study, 51 (45.5%) livestock owners were using acaricides regularly, and 49 (43.8%) had no proper disposal procedures in place for used acaricidal bottles and unused products, instead putting these into general waste streams, including farm drainage systems. Thirty-four (30.4%) livestock owners did not use any acaricides in the year before our visit, but they used them before that. With respect to methods of application, 26 (23.2%) farmers used acaricide systemically, while 34 (30.4%) used topical applications for tick control. Forty-three respondents (38.3%) reported the reinfestation of ticks 30 to 60 days after the use of acaricides. Only 15 (13.4%) respondents considered biosecurity measures as a preventive measure against tick infestation (Table 1).

### 2.4. Zoonotic Perspective

Almost three-quarters of the farmers (71.4%; i.e., 80 respondents) included in this survey practiced manual removal of ticks (bare-handed); out of which 40 (35%) reported tick-bites, where only 32 (28.6%) consulted a physician while 24 (21.4%) opted self-medication. Interestingly, 46 farmers (41.1%) ignored the tick bite, with 27 (24.1%) experiencing restlessness and/or fever-like symptoms, which affected their daily work schedule (Table 1). 

### 2.5. Risk Factors Associated with Tick Prevalence

A Chi-square test was performed to check the association between potential risk factors and tick presence. Significant associations were found between ticks and livestock feeding methods (χ^2^(1) = 112.49, *p* = 0.002), ticks and ignoring their presence (χ^2^(1) = 17.00, *p* = 0.001), and proper plan of acaricidal (using acaricides at least with three month intervals) use and absence of ticks on the animals (χ^2^(1) = 12.09, *p* = 0.001). There was also a statistically significant association between farms using acaricides more than one year ago with the presence of ticks (χ^2^(1) = 15.80, *p* = 0.007), and workers with tick bites (χ^2^(1) = 4.14, *p* = 0.042). Similarly, a statistically significant association was found between tick bites and ticks being removed barehanded (χ^2^(1) = 8.18, *p* = 0.042). Farmers having above a 10th-grade education had sufficient knowledge about tick-borne diseases as a zoonotic risk (χ^2^(1) = 41.10, *p* < 0.001) and were aware that sandy floor was a risk factor for tick presence (χ^2^(1) = 15.01, *p* < 0.001) (Table 2).

**Figure 2:**
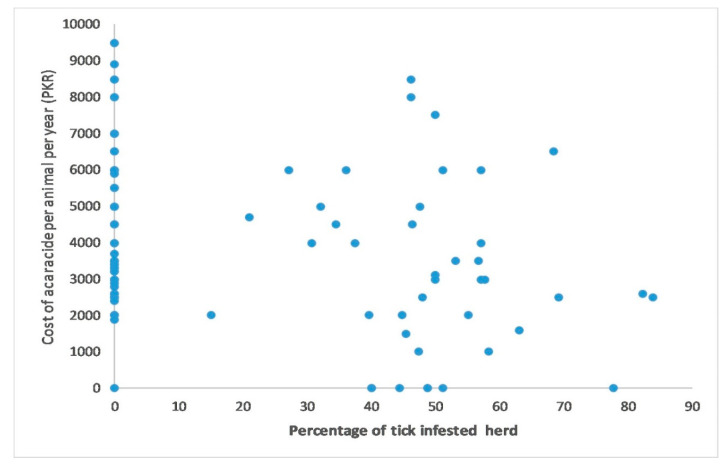
Correlation between total cost (PKR) of acaracide per animal per year on farm and percentage of tick infested animals at the time of survey on farm.

**Figure 3:**
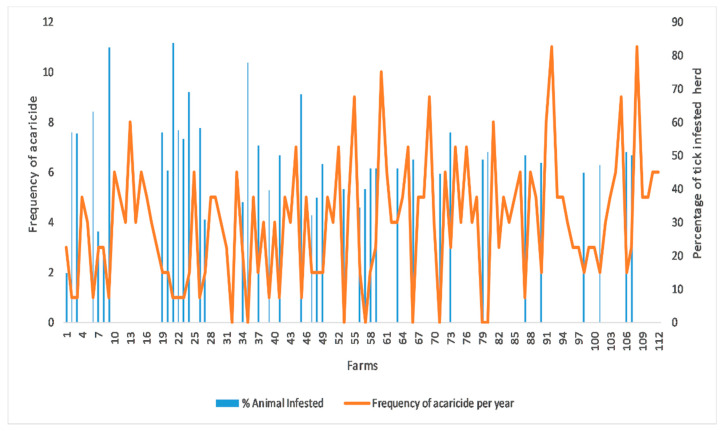
Correlation between frequency of using acaricides on farms and percentage of tick infested animals at the time of survey on farm.

For the quantitative variable analysis, a bivariate correlation test was used to check the correlation between the cost of acaricides per animal per year, which was found very weak but negatively correlated (r(1) = −0.363) with the number of tick-infested animals and statistically significant (*p* < 0.001) (Figure 2). The frequency of using acaricides on farm was found strong but negatively correlated (r(1) = −0.786) with number of tick infested animals on farm and statistically significant (*p* < 0.001) (Figure 3). A binary logistic regression indicated that marshy areas near the farms (OR = 5.29, *p* = 0.001) and dogs infested with ticks (OR = 2.738, *p* = 0.022) were significant predictors for tick presence on the livestock farms. In addition, keeping animals together in herds (OR = 26.085, *p* <0.001) and on sandy floors (OR = 10.57, *p* = 0.001) were potential risk factors for tick presence on the farm. The Hosmer–Lemeshow test indicated value of alpha greater than 0.05, which was non-significant to assure the fitness of our logistic regression model (Table 3).

## 3. Discussion

Ticks and TBDs are among the major veterinary and public health problems world-wide, including Pakistan. In many developing countries, heavy tick infestation and TBDs cause morbidity and mortality in animals and are associated with decreased production of milk, meat, and other livestock products. Among blood-feeding arthropods, ticks transmit more diseases than any other species around the globe, affecting humans, livestock, and companion animals alike [57].

Work elsewhere has reported the high presence of *Haemaphysalis cornupunctata* and *Ha. kashmirensis* in Pakistan, alongside *Hyalomma* tick species, known vectors of *Theileria annulata* [57]. Economic losses due to ticks in this region rapidly escalate when tick prevalence increases, not only through losses to livestock productivity, but also as a result of expenses incurred for acaricide use [58]. Even with the use of acaricide, some farms suffered high tick infestation, which might be due to lack of awareness about the proper use of acaricides (such as diluting the acaricide to save money) and resistance of ticks to the products used [59]. Economic losses caused by ticks are not properly understood in our study region, due to the diversity of ixodid ticks in Pakistan and the lack of national studies focusing on estimation of economic losses attributed to tick infestation [60,61]. A few studies have tried to genetically characterize ticks in this region, but were imitated due to genetic markers (Cox1 and ITS-2) only being able to separate small numbers of tick specimens; further study would be important for those ticks being vectors of potential tick-borne pathogens needing further/different treatments [62]. Moreover, we found that farmers were not adopting biosecurity measures against tick infestation, which might be linked to continuous infestation with ticks, also contributing to acaricide failures in the long-term (by assisting resistance development or promoting reinfestations) and ultimately leading to large, temporally accrued economic losses, even in small herds. 

In the current study, most of the survey respondents were fully reliant on livestock farming for their livelihoods. Most of the farms were heavily infested with ticks, which would have resulted in overall production declines. As there was a strong negative correlation between the frequency of using acaricides with percentage of tick-infested animals, we can consider the frequent and effective use of acaricides as a major reason for difference in the tick prevalence on different farms. The majority of the respondents were unaware of tick infestation as a source of disease transmission in their animals, and only a few knew to contact a qualified veterinarian on observing ticks on their animals. In our study, tick-infested farm dogs played a significant role in enhancing tick presence in livestock, which agrees with previous research elsewhere [63,64]. Similarly, use of herbal or traditional therapeutic approaches as control measures, the lack of proper knowledge on ticks, TBDs, and risk factors, the absence of consultation with a qualified veterinarian or a proper plan of acaricides use, potential promotion of ticks via climate change, sandy floors, and nearby marshy areas due to maximum retainability of moisture and possibility of cracks were possible contributing factors for infestation aligned with previous studies [65]. Farmers in this study reported the summer season as the most high-risk period for tick infestation, which is also consistent with another study conducted in two districts of Punjab; here it was found that the highest tick prevalence of 68.29% and 73.4% (in the Layyah and Muzaffargarh districts, respectively) occurred in July [66]. Our study results related to tick infestation in livestock herds were, however, slightly different (10% lower) from previous studies conducted in Pakistan [66]. We speculate that this might be due to sampling at different times. It is also noted that our results for tick prevalence do align well with another study conducted in Pakistan [67].

The risk of TBPs to humans varies with the tick density, human activities, and occurrence and frequencies of behaviors that cause exposure of people to host-seeking ticks. An improved understanding of human activities and behaviors, specifically in home-based livestock settings, may reduce exposure to tick bites and, ultimately, the risk of TBDs transmission to people [68]. According to a study conducted in Pakistan in 2009, *R. microplus, R. annulatus, Hae. punctata, Hya. marginatum*, and *Hya. anatolicum* were collected from humans (farmers and the general public) [69], highlighting the importance of better managing human-tick interactions in this region. The current study has reported several tick bites in home-based livestock settings, noting that most were simply ignored, which may represent an important public health concern for zoonotic transmission of TBDs. Lack of awareness about the zoonotic potential of TBPs likely facilitates not taking proper action to consult the physician for a possible diagnosis. Our results, and those of others, also show that home-based livestock settings, where people maintain a few animals for their livelihood (representing a major livestock sector in Pakistan), are at a greater risk of getting zoonotic diseases [70,71]. 

Environmental factors and climate change are expected to play a significant role in future patterns of ticks and TBDs, although their relationship has not been yet demonstrated in Pakistan. Ticks are more commonly found in the areas having prolonged extreme temperature ranges, heavy rainfall, and low humidity. Such increase in temperatures and changes in rainfall patterns are highly associated with climate change [71,72,73], which may in turn increase tick infestation where it favors hatching of eggs and tick development. Furthermore, rising temperature may lead to improved survival conditions for ticks and might accelerate their reproduction and lifecycle [74]. In our study, livestock farms located in the high-temperature zones showed notably high tick prevalence, which is aligned with results of a previous study conducted in several districts of Punjab and Canada [75,76,77].

Various acaricides (e.g., products containing Organophosphates such as Trichlorophon) have been used in Pakistan to control ectoparasites. The continuous use of these synthetic pesticides is likely to be restricted, however as their potentially detrimental impact on humans and the environment becomes better understood, as reported in Pakistan, India, and Australia [71,78,79,80], pest control approaches are increasingly assessed within an integrated ‘One Health’ framework. Many of these compounds have a wide range of toxicity levels, are chemically similar to other toxic compounds [81], and can enter milk and meat production chains in the form of residues, through various direct and indirect routes, causing concerns for human health [81]. In our study, livestock owners that were using acaricides to control ticks on their farms were potentially playing a significant role in environmental pollution, with direct exposure of some notably pesticide-sensitive habitats (e.g., aquatic ecosystems) likely to result from observed practices. Respondents had no proper disposal protocols for pesticide bottles, with any left-over acaricides being emptied into drains directed connected to streams and canals located near to the farm. Our results are in agreement with a previously reported study, where more dead organisms were observed in lakes, streams, rivers, and canals located near to farms where insecticides and pesticides were used and handled in an improper way. Hence, we strongly suggest that proper training is needed for the use of acaricides and, importantly, their disposal. 

The current study has conclusively shown that most livestock owners in our study region(s) of Pakistan had little knowledge regarding ticks and TBDs and had not considered an integrated strategy against tick infestation in place. Many risk factors remained neglected on most of the tick-infested farms surveyed due to insufficient awareness, which led to high tick prevalence. Many livestock owners were not hesitant to crush the ticks by hand and commonly reported tick bites, which should be considered as a significant public health concern. Improper disposal of acaricides, including into farm drainage systems, was recorded, a practice that will almost certainly be placing the environment and aquatic life of these regions at risk. The gap in coordination and communication between livestock owners and the Livestock and Dairy Development Department (Extension wing) should be addressed to reduce the burden of TBDs affecting farmers’ economic status, as well as to protect Pakistan’s natural environment. This could potentially be achieved by accelerating the departmental campaigns to raise awareness of farmers about environmentally friendly approaches to preventing and treating tick infestations and promoting healthy and productive livestock. Collaborative research would further help in this area, bringing together epidemiologists, ecologists, and microbiologists to further develop One Health approaches to tick/TBDs management for Pakistan’s livestock farmers. 

## 4. Materials and Methods

### 4.1. Study Area

This study was conducted in seven districts of Punjab, Pakistan, and selected districts are based on the dense population of livestock, markets of animals, and animal products like milk, meat, skin, and hides, in these areas. These districts cover 41,520 km^2^, encapsulating almost half of the ruminant population in Punjab. More specifically, our study areas included: Kasur (31.2° N and 74.5° E), part of the Northern irrigated plans where the climate is semi-arid to arid (east to the south-west), with maximum (summer) and minimum (winter) temperatures of 39.5 °C and 6.2 °C, respectively, and average annual rainfall of 300–500 mm in the east and 200–300 mm in the south-west [21]; Gujranwala (32.18° N, 74.19° E), part of the agro-ecological zone known as ‘Barani’ (rain-fed), its Southwest part is semi-arid and hot, the maximum temperature in summer is 38 °C and 4–7 °C in winter, mean monthly rainfall in summer is 85 mm and 30–45 mm in winter; Sheikhupura (31.7° N, 73.9° E), which is part of the Northern irrigated plans with a maximum (summer), and minimum (winter) [21] temperature is 39 °C and 5 °C, respectively, and an average rainfall of 635 mm; Khushab (32.3° N, 72.5° E), with maximum (summer) and minimum (winter) [21] temperatures of 42 °C and 12 °C, respectively, and average monthly rainfall of 45 mm (summer) and 10–25 mm (winter); Vehari (30.04° N, 72.34° E), with a maximum (summer) and minimum (winter) [21] temperature of 45 to 28 °C and 21 and 5 °C, respectively, and mean annual rainfall of 300–500 mm in the east and 200–300 mm in the south-west; Bahawalnagar (30.0° N, 72.24° E); and Muzaffargarh (30.07° N, 71.18° E), with maximum (summer) and minimum (winter) [21] temperatures of 54 °C and 1–5 °C, respectively, and an average rainfall of circa 127 mm. 

### 4.2. Data Collection

Livestock owners with ruminant herds ranging from 10 to 50 animals were included in the study. Farms surveyed were selected based on operational convenience and willingness to participate. A total of 112 livestock owners were visited, and face-to-face interviews were conducted to collect the information required, based on a structured questionnaire that was organized and prepared in English (see Table 1). However, interviews were delivered in local languages (Urdu, Punjabi, Saraiki) after translation, to maximize the accuracy of responses and minimize any confusion concerning the terminology used. 

In terms of livestock species covered by the survey, cattle breeds (Sahiwal, Friesian, Cross Friesian, Cholistani, Dajjal, Dhanni, and crossbred cattle), buffalo breeds (Nili Ravi, Kundi, and crossbred buffalo), sheep breeds (Kajli, Thali, Sipli, Lohi, and crossbred sheep), and goat breeds (Beetal, Makhi Cheeni, Rajan Puri, Teddi, Nachi, and crossbred goats) were all found in our studied districts. 

### 4.3. Statistical Analysis

All data collected in the form of questionnaires were stored in an excel file (Microsoft Excel 2016). Later, the data were entered in the SPSS (Statistical Package for the Social Sciences) version 25.0. The data were checked and cleaned, and descriptive frequencies were calculated to know the knowledge and practices of livestock owners in selected districts of Punjab, Pakistan. Chi-square test was used to analyze the association of the tick infestation with all other possible risk factors asked and observed during data collection. A CSV was created and imported in open source R software version 3.2.3 and all variables for which maximum association was found in Chi-square test were considered for the logistic regression model to check the contribution of these variables towards tick infestation. The Hosmer–Lemeshow test was performed for the goodness of fit for logistic regression model.

## Figures and Tables

**Table 1:** Survey questions and frequencies of responses provided by the livestock farmers.

Questions	Responses	Frequencies	Percentages
Do you keep your animals together?	Yes	53	47.3
	No	59	52.7
Is there a marshy area near your farm?	Yes	29	25.9
	No	83	74.1
Where do you keep animals?	At home	47	42
	On-farm	65	58
How external staff work on the farm?	Permanently	60	53.6
	Temporary	52	46.4
What animal feeding method do you use?	Stall feeding	28	25
	grazing	24	21.4
	Mixed	60	53.6
Have you heard before about tick infestation?	Yes	64	57.1
	No	48	42.9
Are there dogs on-the farm infested with ticks?	Yes	49	43.8
	No	58	51.8
	No dog presence	5	4.5
Do you know about TBDs as a zoonotic risk factor?	Yes	46	41.1
	No	66	58.9
Were you aware that sandy floor is a risk factor for tick presence?	Yes	53	47.3
	No	59	52.7
In your view, which season is highest risk for tick infestation?	Summer	45	40.2
	Winter	13	11.6
	Spring	37	33
	Autumn	17	15.2
When tick infestation is observed, what do you do?	Call vet	32	28.6
	Herbal/traditional treatment	39	34.8
	Sold their animals to reduce tick infestation	17	15.2
	Ignore	24	21.4
Do you have proper plan for acaricide use?	Yes	51	45.5
	No	61	54.5
Do you have proper disposal procedures or place for used acaricide bottles?	Yes	2	1.8
	No	49	43.8
	Don’t know	61	54.5
When was the last time you used acaricide?	Not more than 2 months ago	17	15.2
	Not more than 4 months ago	16	14.3
	Not more than 6 months ago	19	17
	More than 6 months ago, but less than 1 year ago	18	16.1
	More than a year ago	34	30.4
	Never	8	7.1
What is your method of using acaricides?	Injection	26	23.2
	Topical	34	30.4
	Both	44	39.3
	No acaricidal use	8	7.1
After how much time of using acaricides do you observe ticks at your farm?	Within months	32	28.6
	30 to 60 days later	64	57.1
	60 to 90 days later	15	13.4
	90 to 120 days later	1	0.9
Have you seen milk reductions because of ticks?	Yes	85	75.9
	No	27	24.1
Have ticks affected your animals’ growth?	Yes	69	61.6
	No	43	38.4
Best strategy to prevent tick infestation?	Biosecurity measures	15	13.4
	Acaricide use	54	48.2
	No (cannot be prevented)	37	33
	Do not know)	6	5.4
What types of floor do you have?	Sandy	46	41.1
	Concreted	66	58.9
How do you remove ticks when found on animals?	Gloves	9	8
	Hands	80	71.4
	Forceps	13	11.6
	Tweezers	10	8.9
Have workers been bitten by ticks?	Yes	40	35.7
	No	72	64.3
What actions do you take for human tick bites?	Consult physician	32	28.6
	Self-medication	24	21.4
	Consult spiritual healer	10	8.9
	Ignore bite	46	41.1
Have you experienced and fever-like symptoms after tick bites?	Yes	27	24.1
	No	85	75.9
Has your capacity to work been affected by tick bites?	Yes	27	24.1
	No	13	11.6
	Not been bitten	72	64.3
Have you observed ticks at home?	Yes	91	81.3
	No	21	18.8
What measures do you take if ticks found at home?	Spray	11	9.8
	Powder pesticide	43	38.4
	Remove ticks physically	31	27.7
	Insect repellent on ticks	12	10.7
	Vaseline oil or alcohol	7	6.3
	Ignore	8	7.1
Average acaricides’ cost per animal per year among 104 respondents	4535 PKR	Range	(1000–9500)
Average Frequency of using acaricides on farm per year among 104 respondents	4	Range	(1–11)

**Table 2:** Chi-square analysis of associated risk factors.

Questions	χ^2^	*p* Value
Responses with reference to presence of ticks		
Mixed type of feeding method	12.49	0.002
Ignore the animal upon tick infestation	17.00	0.001
No proper plan of acaricide use	12.09	0.001
Acaricide used last time more than a year ago	15.8	0.007
Unawareness about biosecurity measures as a tick infestation	16.8	0.001
Workers bitten by ticks	4.14	0.042
Responses with reference to knowledge about ticks and tick-borne diseases		
Know about zoonotic nature of tick infestation	41.1	<0.001
Know about sandy floor type as a risk factor for tick infestation	15.01	0.001
Ignoring the human tick bites	20.57	0.002
Responses with reference to removal of ticks		
Worker bitten by ticks	8.18	0.042

**Table 3:** Binary logistic regression analysis of associated risk factors retained in the final model.

Predicting Factors	B	*p* Value	OR	Upper and Lower Value
Responses with reference to presence of ticks				
Animals kept together	3.26	<0.001	26.085	90.74; 7.49
Sandy type of floor	2.35	0.001	10.572	70.04; 2.39
Marshy area near farm	1.667	0.001	5.29	13.55; 2.06
Dog infested with ticks on farm	10.572	0.002	2.73	6.49; 1.15

## Data Availability

The dataset generated for this study is available from the corresponding author upon request.

## References

[1] Ramzan M., Unsar N., Syed H., Ghulam M., Alamgir A. (2018). Knowledge, attitude and practices of herdsmen about ticks and tick–borne diseases in district Multan. Pak. Entomol..

[2] Furman D.P., Loomis E.C. (1984). The Ticks of California (Acari: Ixodida).

[3] Kabir M., Mondal M., Eliyas M., Mannan M., Hashem M., Debnath N., Miazi O., Kashem M., Islam M., Elahi M. (2011). An epidemiological survey on investigation of tick infestation in cattle at Chittagong District, Bangladesh. Afr. J. Microbiol. Res..

[4] Anderson J.F., Magnarelli L.A. (2008). Biology of ticks. Infect. Dis. Clin. N. Am..

[5] Jongejan F., Uilenberg G. (2004). The global importance of ticks. Parasitol. Camb..

[6] Sajid M.S., Iqbal Z., Khan M.N., Muhammad G. (2008). Point prevalence of hard ticks (Ixodids) infesting domestic ruminants of lower Punjab, Pakistan. Int. J. Agric. Biol..

[7] Iqbal A., Sajid M.S., Khan M.N., Khan M.K. (2013). Frequency distribution of hard ticks (Acari: Ixodidae) infesting bubaline population of district Toba Tek Singh, Punjab, Pakistan. Parasitol. Res..

[8] Rehman A., Nijhof A.M., Sauter-Louis C., Schauer B., Staubach C., Conraths F.J. (2017). Distribution of ticks infesting ruminants and risk factors associated with high tick prevalence in livestock farms in the semi-arid and arid agro-ecological zones of Pakistan. Parasites Vectors.

[9] Ghafar A., Gasser R.B., Rashid I., Ghafoor A., Jabbar A. (2020). Exploring the prevalence and diversity of bovine ticks in five agro-ecological zones of Pakistan using phenetic and genetic tools. Ticks Tick-Borne Dis..

[10] Jabbar A., Abbas T., Saddiqi H.A., Qamar M.F., Gasser R.B. (2015). Tick-borne diseases of bovines in Pakistan: Major scope for future research and improved control. Parasites Vectors.

[11] Karim S., Budachetri K., Mukherjee N., Williams J., Kausar A., Hassan M.J., Adamson S., Dowd S.E., Apanskevich D., Arijo A. (2017). A study of ticks and tick-borne livestock pathogens in Pakistan. PLoS Negl. Trop. Dis..

[12] Uilenberg G. (1992). Veterinary Significance of Ticks and Tick-Borne Diseases, Tick Vector Biology.

[13] De la Fuente J., Estrada-Pena A., Venzal J.M., Kocan K.M., Sonenshine D.E. (2008). Overview: Ticks as vectors of pathogens that cause disease in humans and animals. Front. Biosci..

[14] Uilenberg G. (1997). General review of tick-borne diseases of sheep and goats world-wide. Parassitologia.

[15] Ahmed J.S., Luo J., Schnittger L., Seitzer U., Jongejan F., Yin H. (2006). Phylogenetic position of small-ruminant infecting piroplasms. Ann. N. Y. Acad. Sci..

[16] Carter S.D., Surtees R., Walter C.T., Ariza A., Bergeron É., Nichol S.T., Hiscox J.A., Edwards T.A., Barr J.N. (2012). Structure, function, and evolution of the Crimean-Congo hemorrhagic fever virus nucleocapsid protein. J. Virol..

[17] Charrel R.N., Attoui H., Butenko A., Clegg J., Deubel V., Frolova T., Gould E., Gritsun T., Heinz F., Labuda M. (2004). Tick-borne virus diseases of human interest in Europe. Clin. Microbiol. Infect..

[18] Kasi K.K., Sas M.A., Sauter-Louis C., von Arnim F., Gethmann J.M., Schulz A., Wernike K., Groschup M.H., Conraths F.J. (2020). Epidemiological investigations of Crimean-Congo haemorrhagic fever virus infection in sheep and goats in Balochistan, Pakistan. Ticks Tick-Borne Dis..

[19] Kasi K.K., von Arnim F., Schulz A., Rehman A., Chudhary A., Oneeb M., Sas M.A., Jamil T., Maksimov P., Sauter-Louis C. (2020). Crimean-Congo haemorrhagic fever virus in ticks collected from livestock in Balochistan, Pakistan. Transbound. Emerg. Dis..

[20] Nuttall P., Labuda M. (2003). Dynamics of infection in tick vectors and at the tick-host interface. Adv. Virus Res..

[21] Parola P., Paddock C.D., Raoult D. (2005). Tick-borne rickettsioses around the world: Emerging diseases challenging old concepts. Clin. Microbiol. Rev..

[22] Daniel M., Benes C., Danielová V., Kriz B. (2011). Sixty years of research of tick-borne encephalitis—A basis of the current knowledge of the epidemiological situation in Central Europe. Epidemiol. Mikrobiol. Imunol. Cas. Spol. Epidemiol. Mikrobiol. Ceske Lek. Spol. JE Purkyne.

[23] Süss J. (2011). Tick-borne encephalitis 2010: Epidemiology, risk areas, and virus strains in Europe and Asia—An overview. Ticks Tick-Borne Dis..

[24] Maltezou H.C., Papa A. (2011). Crimean-Congo hemorrhagic fever: Epidemiological trends and controversies in treatment. BMC Med..

[25] Stanek G., Wormser G.P., Gray J., Strle F. (2012). Lyme borreliosis. Lancet.

[26] Amicizia D., Domnich A., Panatto D., Lai P.L., Cristina M.L., Avio U., Gasparini R. (2013). Epidemiology of tick-borne encephalitis (TBE) in Europe and its prevention by available vaccines. Hum. Vaccines Immunother..

[27] Petrulionienė A., Radzišauskienė D., Ambrozaitis A., Čaplinskas S., Paulauskas A., Venalis A. (2020). Epidemiology of Lyme Disease in a Highly Endemic European Zone. Medicina.

[28] Parola P., Paddock C.D., Socolovschi C., Labruna M.B., Mediannikov O., Kernif T., Abdad M.Y., Stenos J., Bitam I., Fournier P.-E. (2013). Update on tick-borne rickettsioses around the world: A geographic approach. Clin. Microbiol. Rev..

[29] Moneret-Vautrin D.A., Beaudouin E., Kanny G., Guérin L., Roche J.-F. (1998). Anaphylactic shock caused by ticks (Ixodes ricinus). J. Allergy Clin. Immunol..

[30] Fernández-Soto P., Davila I., Laffond E., Lorente F., Encinas-Grandes A., Pérez-Sánchez R. (2001). Tick-bite-induced anaphylaxis in Spain. Ann. Trop. Med. Parasitol..

[31] Castelli E., Caputo V., Morello V., Tomasino R.M. (2008). Local reactions to tick bites. Am. J. Dermatopathol..

[32] Buczek W., Buczek A.M., Bartosik K., Buczek A. (2020). Comparison of Skin Lesions Caused by Ixodes ricinus Ticks and Lipoptena cervi Deer Keds Infesting Humans in the Natural Environment. Int. J. Environ. Res. Public Health.

[33] George J., Pound J., Davey R. (2004). Chemical control of ticks on cattle and the resistance of these parasites to acaricides. Parasitology.

[34] Abbas R.Z., Zaman M.A., Colwell D.D., Gilleard J., Iqbal Z. (2014). Acaricide resistance in cattle ticks and approaches to its management: The state of play. Vet. Parasitol..

[35] Ray D.E., Ray D., Forshaw P.J. (2000). Pyrethroid insecticides: Poisoning syndromes, synergies, and therapy. J. Toxicol. Clin. Toxicol..

[36] Litovitz T.L., Klein-Schwartz W., Rodgers G.C., Cobaugh D.J., Youniss J., Omslaer J.C., May M.E., Woolf A.D., Benson B.E. (2002). 2001 Annual report of the American Association of Poison Control Centers toxic exposure surveillance system. Am. J. Emerg. Med..

[37] Dahlgren L., Johnson R.M., Siegfried B.D., Ellis M.D. (2012). Comparative toxicity of acaricides to honey bee (Hymenoptera: Apidae) workers and queens. J. Econ. Entomol..

[38] De Meneghi D., Stachurski F., Adakal H. (2016). Experiences in tick control by acaricide in the traditional cattle sector in Zambia and Burkina Faso: Possible environmental and public health implications. Front. Public Health.

[39] Caporale D.A., Rich S.M., Spielman A., Telford S.R., Kocher T.D. (1995). Discriminating between Ixodes ticks by means of mitochondrial DNA sequences. Mol. Phylogenetics Evol..

[40] Walker A.R. (2003). Ticks of Domestic Animals in Africa: A Guide to Identification of Species.

[41] Parola P., Socolovschi C., Jeanjean L., Bitam I., Fournier P.-E., Sotto A., Labauge P., Raoult D. (2008). Warmer weather linked to tick attack and emergence of severe rickettsioses. PLoS Negl. Trop. Dis..

[42] Süss J., Klaus C., Gerstengarbe F.W., Werner P.C. (2008). What makes ticks tick? Climate change, ticks, and tick-borne diseases. J. Travel Med..

[43] Jaenson T.G., Jaenson D.G., Eisen L., Petersson E., Lindgren E. (2012). Changes in the geographical distribution and abundance of the tick Ixodes ricinus during the past 30 years in Sweden. Parasites Vectors.

[44] Mysterud A., Stigum V.M., Seland I.V., Herland A., Easterday W.R., Jore S., Østerås O., Viljugrein H. (2018). Tick abundance, pathogen prevalence, and disease incidence in two contrasting regions at the northern distribution range of Europe. Parasites Vectors.

[45] Jahfari S., Hofhuis A., Fonville M., van der Giessen J., van Pelt W., Sprong H. (2016). Molecular detection of tick-borne pathogens in humans with tick bites and erythema migrans, in the Netherlands. PLoS Negl. Trop. Dis..

[46] Černý J., Lynn G., Hrnková J., Golovchenko M., Rudenko N., Grubhoffer L. (2020). Management Options for Ixodes ricinus-Associated Pathogens: A Review of Prevention Strategies. Int. J. Environ. Res. Public Health.

[47] Buczek A., Bartosik K., Wisniowski L., Tomasiewicz K. (2013). Changes in population abundance of adult Dermacentor reticulatus (Acari: Amblyommidae) in long-term investigations in eastern Poland. Ann. Agric. Environ. Med..

[48] Hasle G. (2013). Transport of ixodid ticks and tick-borne pathogens by migratory birds. Front. Cell. Infect. Microbiol..

[49] Buczek A.M., Buczek W., Buczek A., Bartosik K. (2020). The Potential Role of Migratory Birds in the Rapid Spread of Ticks and Tick-Borne Pathogens in the Changing Climatic and Environmental Conditions in Europe. Int. J. Environ. Res. Public Health.

[50] Rafiq N., Kakar A., Ghani A., Iqbal A., Achakzai W.M., Sadozai S., Shafiq M., Mengal M.A. (2017). Ixodid ticks (Arachnida: Acari) prevalence associated with risk factors in the bovine host in District Quetta, Balochistan. Pak. J. Zool..

[51] Ahmed A., Saqlain M., Tanveer M., Tahir A.H., Ud-Din F., Shinwari M.I., Khan G.M., Anwer N. (2021). Knowledge, attitude and perceptions about Crimean Congo Haemorrhagic Fever (CCHF) among occupationally high-risk healthcare professionals of Pakistan. BMC Infect. Dis..

[52] Ali Z., Kumar R., Ahmed J., Ghaffar A., Mureed S. (2013). Knowledge, attitude and practice of Crimean-Congo hemorrhagic fever among rural population of Baluchistan, Pakistan. A public health nutritional assessment of elderly in Islamabad: A mixed method study. J. Public Health.

[53] Shadick N.A., Daltroy L.H., Phillips C.B., Liang U.S., Liang M.H. (1997). Determinants of tick-avoidance behaviors in an endemic area for Lyme disease. Am. J. Prev. Med..

[54] Riccò M., Gualerzi G., Ranzieri S., Ferraro P., Bragazzi N.L. (2020). Knowledge, attitudes, practices (KAP) of Italian occupational physicians towards tick borne encephalitis. Trop. Med. Infect. Dis..

[55] Noden B.H., Garner K.D., Lalman D., Talley J.L. (2020). Knowledge, Attitudes, and Practices Regarding Ticks, Tick-Borne Pathogens, and Tick Prevention among Beef Producers in Oklahoma. Southwest. Entomol..

[56] Buczek A., Pilch J., Buczek W. (2020). Tick preventive behaviors and practices adopted by medical students from Poland, Germany, and Thailand in relation to socio-demographic conditions and their knowledge of ticks and tick-borne diseases. Insects.

[57] Hoogstraal H., Varma M. (1962). Haemaphysalis cornupunctata sp. n. and H. kashmirensis sp. n. from Kashmir, with Notes on H. sundrai Sharif and H. sewelli Sharif of India and Pakistan (Ixodoidea, Ixodidae). J. Parasitol..

[58] Batool M., Nasir S., Rafique A., Yousaf I., Yousaf M. (2019). Prevalence of tick infestation in farm animals from Punjab, Pakistan. Pak. Vet. J..

[59] Ghosh S., Kumar R., Nagar G., Kumar S., Sharma A.K., Srivastava A., Kumar S., Kumar K.A., Saravanan B. (2015). Survey of acaricides resistance status of Rhipiciphalus (Boophilus) microplus collected from selected places of Bihar, an eastern state of India. Ticks Tick-Borne Dis..

[60] Roy B.C., Estrada-Peña A., Krücken J., Rehman A., Nijhof A.M. (2018). Morphological and phylogenetic analyses of Rhipicephalus microplus ticks from Bangladesh, Pakistan and Myanmar. Ticks Tick-Borne Dis..

[61] Sands A.F., Apanaskevich D.A., Matthee S., Horak I.G., Harrison A., Karim S., Mohammad M.K., Mumcuoglu K.Y., Rajakaruna R.S., Santos-Silva M.M. (2017). Effects of tectonics and large scale climatic changes on the evolutionary history of Hyalomma ticks. Mol. Phylogenet. Evol..

[62] Irshad N., Qayyum M., Hussain M., Khan M.Q. (2010). Prevalence of tick infestation and theileriosis in sheep and goats. Pak. Vet. J..

[63] Cromley E.K., Cartter M.L., Mrozinski R.D., Ertel S.-H. (1998). Residential setting as a risk factor for Lyme disease in a hyperendemic region. Am. J. Epidemiol..

[64] Steere A.C., Broderick T.F., Malawista S.E. (1978). Erythema chronicum migrans and lyme arthritis: Epidemiologic evidence for a tick vector1. Am. J. Epidemiol..

[65] Riaz M., Ullah M. (2017). Epidemiological survey on diversity and seasonal distribution of hard ticks in sheep and goats in Multan, Pakistan. J. Biodivers. Environ. Sci..

[66] Sajid M.S., Iqbal Z., Khan M.N., Muhammad G., Khan M.K. (2009). Prevalence and associated risk factors for bovine tick infestation in two districts of lower Punjab, Pakistan. Prev. Vet. Med..

[67] Ramzan M., Khan M.S., Avais M., Khan J.A., Pervez K., Shahzad W. (2008). Prevalence of ecto parasites and comparative efficacy of different drugs against tick infestation in cattle. J. Anim. Pl. Sci..

[68] Nelson C.A., Saha S., Kugeler K.J., Delorey M.J., Shankar M.B., Hinckley A.F., Mead P.S. (2015). Incidence of clinician-diagnosed Lyme disease, United States, 2005–2010. Emerg. Infect. Dis..

[69] Ali A., Khan M.A., Zahid H., Yaseen P.M., Khan M.Q., Nawab J., Rehman Z.U., Ateeq M., Khan S., Ibrahim M. (2019). Seasonal Dynamics, Record of Ticks Infesting Humans, Wild and Domestic Animals and Molecular Phylogeny of Rhipicephalus microplus in Khyber Pakhtunkhwa Pakistan. Front. Physiol..

[70] Eisen R.J., Eisen L., Beard C.B. (2016). County-scale distribution of Ixodes scapularis and Ixodes pacificus (Acari: Ixodidae) in the continental United States. J. Med Entomol..

[71] Baker J. Resistance to ixodicides by ticks in Africa south of the Equator with some thoughts on tick control in this area. Proceedings of the Tick Borne Diseases and their Vectors, Proceedings of an International Conference.

[72] Ashraf S., Parveen A., Asif M., Awais M.M., Khan A., Aktas M., Ozubek S., Alanazi A.D., Alyousif M.S., Iqbal F. (2020). A Report on the Tick Burden, Molecular Detection and Phylogenetic Analysis of Anaplasma Marginale in the Blood Samples of Cattle Collected from District Layyah in Punjab (Pakistan). Curr. Microbiol..

[73] Warren F.J., Lemmen D.S. (2014). Canada in a Changing Climate: Sector Perspectives on Impacts and Adaptation.

[74] Ogden N.H., Lindsay L.R. (2016). Effects of climate and climate change on vectors and vector-borne diseases: Ticks are different. Trends Parasitol..

[75] Atif F., Khan M., Iqbal H., Ali Z., Ullah S. (2012). Prevalence of cattle tick infestation in three districts of the Punjab, Pakistan. Pak. J. Sci..

[76] Gasmi S., Bouchard C., Ogden N.H., Adam-Poupart A., Pelcat Y., Rees E.E., Milord F., Leighton P.A., Lindsay R.L., Koffi J.K. (2018). Evidence for increasing densities and geographic ranges of tick species of public health significance other than Ixodes scapularis in Québec, Canada. PLoS ONE.

[77] Ogden N., Bigras-Poulin M., O’callaghan C., Barker I., Lindsay L., Maarouf A., Smoyer-Tomic K., Waltner-Toews D., Charron D. (2005). A dynamic population model to investigate effects of climate on geographic range and seasonality of the tick Ixodes scapularis. Int. J. Parasitol..

[78] Sajid M.S., Iqbal Z., Khan M.N., Muhammad G., Needham G., Khan M.K. (2011). Prevalence, associated determinants, and in vivo chemotherapeutic control of hard ticks (Acari: Ixodidae) infesting domestic goats (Capra hircus) of lower Punjab, Pakistan. Parasitol. Res..

[79] World Health Organization, Food and Agriculture Organization of the United Nations (FAO) (1984). Data Sheet on Pesticides No. 65: Bis (Tributyltin) Oxide.

[80] Roulston W., Wharton R., Schnitzerling H., Sutherst R., Sullivan N. (1971). Mixtures of chlorphenamidine with other acaricides for the control of organophosphorus-resistant strains of cattle tick Boophilus microplus. Aust. Vet. J..

[81] Kunz S., Kemp D. (1994). Insecticides and acaricides: Resistance and environmental impact. Rev. Sci. Tech. (Int. Off. Epizoot.).

