# Correction: Hussain et al. An Epidemiological Survey Regarding Ticks and Tick-Borne Diseases among Livestock Owners in Punjab, Pakistan: A One Health Context. *Pathogens* 2021, *10*, 361

**DOI:** 10.3390/pathogens13121093

**Published:** 2024-12-11

**Authors:** Sabir Hussain, Abrar Hussain, Jeffery Ho, Jun Li, David George, Abdul Rehman, Jehan Zeb, Olivier Sparagano

**Affiliations:** 1 Department of Infectious Diseases and Public Health, Jockey Club College of Veterinary Medicine and Life Sciences, City University of Hong Kong, Kowloon, Hong Kong, China; HO.jeff@connect.PolyU.hk (J.H.); jun.li@cityu.edu.hk (J.L.); 2 Department of Epidemiology and Public Health, University of Veterinary and Animal Sciences, Lahore 54600, Pakistan; abrar.arid@gmail.com (A.H.); abdul.rehman@uvas.edu.pk (A.R.); 3 School of Data Science, City University of Hong Kong, Kowloon, Hong Kong, China; 4 School of Natural and Environmental Sciences, Agriculture Building, Newcastle University, Newcastle upon Tyne NE1 7RU, UK; david.george1@newcastle.ac.uk; 5 Department of Zoology, Abdul Wali Khan University Mardan, Mardan 23200, Pakistan; zebjehan2012@gmail.com

There was an error in the original publication [1]. Human ethics approval was missing due to an unintentional mistake. A correction has been made to “Institutional Review Board Statement”.

**Institutional Review Board Statement:** This survey-based study was conducted according to all relevant Animal Welfare Acts and to the guidelines of the Declaration of Helsinki and approved by the JCC Human Ethics Committee of the City University of Hong Kong (protocol code JCC2021ay004, 7 January 2021). The consent form was translated into the relevant local languages (Urdu, Punjabi, Saraiki). All participants and their attendees were briefed about the purpose of the research, interview, and questions; the voluntary participation; and the data anonymity of the study.

The authors state that the scientific conclusions are unaffected. This correction was approved by the Academic Editor. The original publication has also been updated.
